# Estimating the prevalence of stress among Indian students during the COVID-19 pandemic: A cross-sectional study from India

**DOI:** 10.1016/j.jtumed.2020.12.012

**Published:** 2021-01-18

**Authors:** Bijoy Chhetri, Lalit M. Goyal, Mamta Mittal, Gopi Battineni

**Affiliations:** a Department of CE, JC Bose University of Science and Technology, YMCA, Faridabad, India; b Department of CSE, G B Pant Government Engineering College, Okhla, New Delhi, India; c Department of Medical Informatics, School of Medicinal and Health Products Sciences, University of Camerino, Camerino, Italy

**Keywords:** وباء كوفيد-١٩, الإجهاد النفسي, الطلاب الهنود, التركيبة السكانية, أخذ عينات كرة الثلج, COVID-19 epidemic, Demographics, Indian students, Psychological stress, Snowball sampling

## Abstract

**Objective:**

Since December 2019, the COVID-19 pandemic has posed a substantial threat with its associated high mortality, infection, and risk of psychological stress. A large number of students are affected because of a prolonged break from academic activities and staying at home. The focus of this study is to understand the stress levels of Indian students, any psychological imbalances, and their major hurdles during the COVID-19 lockdown.

**Methods:**

Using a snowball sampling method, an online survey of the Perceived Stress Scale (PSS) was conducted on students across India. Along with their demographic details, the participants also reported their study patterns and challenges during their confinement period. The statistical scores for the responses were calculated and the demographic variables analysed. The levels indicated by the PSS were compared, and variance and regression analyses were performed.

**Results:**

We observed that students were generally stressed during lockdown and the pandemic. Females (mean = 3.03) were more stressed than males (mean = 2.61) as they were constantly under pressure because of stressful life events (OR = 0.752, 95% CI = 2.425–310.642) and apprehensive about their studies (RII = 0.67, OR = 2.168, 95% CI = 0.332–6.691).

**Conclusion:**

During the pandemic, students’ mental health needs to be continually monitored as they are stressed owing to fear as well as about their studies and future careers.

## الملخص

**أهداف البحث:**

منذ ديسمبر ٢٠١٩، شكل وباء كوفيد-١٩ تهديدا كبيرا مع ارتفاع عدد الوفيات، والعدوى، ومخاطر الإجهاد النفسي. تأثر عدد كبير من الطلبة بسبب الاستراحة الطويلة من النشاطات الأكاديمية والجلوس في البيت. محور هذه الدراسة هو فهم مستوى التوتر عند الطلاب في الهند، واختلال التوازن النفسي، والعقبة الرئيسة خلال الإغلاق الكامل بسبب كوفيد-١٩.

**طرق البحث:**

باستخدام طريقة أخذ عينات كرة الثلج. تم إجراء مسح عبر الإنترنت لمقياس الإجهاد المتصور على الطلاب عبر الهند. مع التفاصيل الديموغرافية، سجل المشاركون أيضا عن أنماط الدراسة والتحديات خلال فترة الإغلاق. تم حساب الدرجات الإحصائية للردود وتحليل المتغيرات الديموغرافية. تم مقارنة مستوى مقياس الإجهاد وأجريت تحليلات التباين والانحدار.

**النتائج:**

لاحظنا أن الطلاب كانوا خلال الإغلاق والجائحة متوترين بشكل عام. وكان الإناث (المتوسط =٣.٠٣) متوترات أكثر من الذكور (المتوسط =٢.٦١) حيث إنهم كانوا تحت الضغط باستمرار بسبب أحداث الحياة المجهدة.

**الاستنتاجات:**

خلال الجائحة، يجب مراقبة صحة الطلاب العقلية بشكل مستمر لأنهم متوترون من الخوف حول دراستهم ومستقبلهم الوظيفي.

## Introduction

The novel coronavirus (that causes COVID-19) has for many months been a global phenomenon. With its rapid spread rate, it has caused major disruptions to the livelihoods of people worldwide.^1^^,^^2^ As of 30 November 2020, the pandemic had caused nearly 1.46 million deaths of 63.1 million confirmed cases, and put people under tremendous psychological pressure.^3^ Specifically, isolation, engaging in online classes, frequent network failure, and peer and parental pressure have added to students’ perceived stress. As expected, the pandemic has influenced the psychological health of students worldwide.^4^ Therefore, a sufficient number of the around 3.4 million Indian students enrolled in higher education may be a victim of such distress.

With more than 9.4 million confirmed cases including more than 140,000 deaths, India is becoming the second-highest country hit by the pandemic after the USA. Thus, to control the spread of COVID-19, educational institutions like colleges and universities are not opening to students, thereby obstructing regular educational activities. Students felt discontinuity during the lockdown even though the state government issued various educational policies on conducting virtual teaching sessions. Lack of routine student engagement with their university or college resulted in isolation, social media addiction, and no physical activities, which lead to psychological imbalance.

Reportedly, the general public, patients, medical staff, children, and older adults are highly vulnerable to psychological health issues during an epidemic.^5^ Some studies highlighted stress among students, although many global universities are anticipated to have implemented serious measures to prevent stress among staff and students.^6^^,^^7^ Pandemics like COVID-19 not only affect daily life activities, but also create additional delays in academic activities, which are positively correlated with stress and students’ level of anxiety.^7^

Many countries have encouraged medical students to collaborate with national health workers in this prevailing situation. These medical students are under high pressure and stress because of direct contact with infected people.^8^ Because of fear, stress, and anxiety added by COVID-19 infection, students are at high risk of experiencing psychological issues.^9^ A study by Odriozola P. et al. (2020) confirmed the stress and other severe psychological distress due to the COVID-19 outbreak among students and workers in Spanish universities.^10^ However, no significant study has highlighted the mental health of Indian students during the current pandemic.

Common individual behavioural effects like anxiety, stress, depression, anger, and post-traumatic stress are socially available disorders affecting people globally. Additional attributes concerning students' like substance abuse, violation of guidelines, peer pressure, and technical glitches during self-learning activities also affect their psychological behaviour. Thus, the focus of this study was to bridge the gap between understanding students’ stress parameters when academic activities are limited and they are left in isolation. The study is based on the 10-item Perceived Stress Scale (PSS), but also considers other factors to proactively identify the stress level of the student fraternity and reason therefor.

## Materials and Methods

### Participants

In this study, a survey on the student population was conducted during the period of home isolation due to the closure of universities and schools. A cross-sectional prevalence study using a simple and convenient snowball sampling method was performed with a structured online questionnaire based on PSS. The authors developed a self-constructed questionnaire to retrieve information on attitudes towards stress caused by COVID-19. Information on basic demographic data, stress prevalence, and attitude towards the stressful event was collected using a Google form circulated through social media platforms like Facebook, WhatsApp, and email. When participants agreed and submitted their response, self-consent was acknowledged. The criteria for participation in the survey were that participants had to be aged at least 15 years and understand English well. Students who did not meet these criteria were excluded. We randomly selected five local colleges in the capital of Delhi and surroundings and included students from different provinces. Participants were asked to name their provinces to identify the geographical area in which they were residing when the survey was conducted. The online questionnaire was delivered to 1536 students and responses were collected over a period of 30 days.

Once participants submitted their responses using the Google form along with their email address, they were assumed to have given consent to participate in the study. After a month, only 450 (29.3%) students had submitted their responses to the survey. As per the responses received, 70% of the targeted sample did not respond; thus, they were considered to have not consented to participate in the study. Students’ personal information and colleges were kept confidential after receiving their consent to participate.

### Subject screening

The PSS, which is measured on a 10-item Likert scale, was adopted to identify and screen candidates.^11^ This tool has been used to measure the degree of impact on individuals' lives of the current situation of stressful events. Only employing the PSS to measure the level of perceived stress does not clarify whether stress is increasing because of COVID-19 or for other reasons unless separate information is sought regarding attitude towards the pandemic. Therefore, we included a few additional questions to understand students’ attitudes in terms of fear, worry, problems faced during the period, and why such perceived stress is occurring. These responses were correlated with the PSS factors and analysed based on the overall observation.

The PSS includes several direct questions about the current level of experienced stress and perceived psychometric evidence regarding personality and social support.^12^ All questionnaire items are reliable in predicting participants' level of stress. Though the PSS is temporal and its predictive validity may decrease over time, it can be used to determine daily activities, events, and changes in a situation. PSS scores are obtained by reversing responses (e.g., 0 = 4, 1 = 3, 2 = 2, 3 = 1, & 4 = 0) to the four positively stated items (items 4, 5, 7, & 8) and then summing all scale items. The total score of the PSS-10 ranges from 0 to 40, and a higher score indicates a higher level of perceived stress. Some studies have already proven that it is reliable and consistent in terms of internal consistency and test-retest reliability across various trial populations.^13^, ^14^, ^15^ The Cronbach's alpha coefficients range from 0.67 to 0.91.

### Online questionnaire

In addition to the PSS questionnaire, an online self-reported questionnaire was used. The prevalence of stress factors among students was determined though the PSS, but this measurement did not satisfy our need to know the correlation of the level of stress with the current pandemic situation. Therefore, a separate assessment in relation to COVID-19 was performed by seeking additional information from the participants. The authors named this attitude towards COVID-19. The motive was to understand the reason behind the perceived level of stress based on the current pandemic and its effects such as institutional closures, online modes of learning, fear of infection, and so on. The questionnaire included items to elicit information on attitude towards COVID-19, willingness towards e-learning, and major hurdles experienced during the lockdown period, as shown in Table 1. The PSS scale measured the level of stress perceived in terms of low, mild, and high. However, understanding why stress is increasing was examined through the questionnaire based on the attitude parameters regarding the pandemic.

**Table 1:** Self-reported questionnaire to determine student attitude towards the pandemic.

Purpose	Questionnaire Item	Type of Scale
Fear	1. Are you scared/stressed by the COVID-19 pandemic?	Rating Scale (0–4)
Worry	2. Are you worried about your studies during this confinement period and post-opening of the institution?	Rating Scale (0–4)
Problem	3. What was the major hurdle during the COVID-19 lockdown period?	Items (P1: Online Classes, P2: Food, or P3: Self-Management)
WHY?	4. Do you think the above PSS stress (questions 1–10) is due to the following:	Likert scale for items (WHY1: Medical reason, WHY2: Greater vulnerability to stressful life-event elicited depressive symptoms, WHY3: Drug & Alcohol, WHY4: E-learning system, WHY5: Any other)

These parameters were included to correlate participants’ health status with a stressful life due to COVID-19 and the sudden change in educational activities.

### Statistical and factor analysis

Statistical analysis was performed to describe the coefficients to summarise the response data. The mean, median, and modes were measured to indicate the centroid along with a variability test using the standard deviation. The Statistical Package for the Social Sciences (SPSS) version 25 was used for data analysis. The threshold level of parametric significance was p < 0.05. Level of perceived stress was classified as low, mild, and severe.

A factor analysis was performed to identify the positive and negative psychological aspects of the current pandemic situation. Regarding reliability, Cronbach's alpha was higher than 0.7 after deleting a factor that decreased the impact of the inter-item relationship. The Relative Importance Index (RII) was employed for the response variables to rank stress-related items in the self-reported questionnaire on attitude towards COVID-19 and academic setbacks due to the pandemic. In addition, a correlation analysis was conducted to evaluate the relationship between students' problems and the reasons underlying their perceived stress. Along with a logistic regression, a one-way ANOVA was performed to check the variance of socio-demographic details, which was compared with its statistical significance.

## Results

### Demographic characteristics of participants

Table 2 presents the demographic details of participating students. Of the sample, 411 (91.33%) students responded to all questions in the survey. The mean and standard deviation (SD) of their age is 23.87 ± 5.51 years (range 15–33 years). Of 411 students, 362 (88.07%) are aged between 19 and 25 years. In addition, 262 (63.7%) are male and 149 (36.3%) are female. In total, 99% of the students agreed to follow the health guidelines issued by the government, and 98.7% were confined at home. The most participants responded from Sikkim (42%), followed by Delhi (29%), Haryana (12%), and from other states (17%). Furthermore, 358 (87%) reported being interested in attending online academic activities and 372 (90%) refrained from the consumption of licit or illicit drugs during the pandemic.

**Table 2:** Socio-demographic details of participants.

Socio-demographic variables	N	%
**Sex**		
Male	262	63.7
Female	149	36.3
**Age Category**		
15–18 years	35	8.5
19–25 years	362	88.1
26–33 years	14	3.4
**Where are they studying?**		
College	296	72.0
University	104	25.3
School	11	2.7
**State of residence**		
Sikkim	170	41.0
Delhi	122	29.0
Haryana	52	12.0
**Services involved in during lockdown**		
Home confinement	350	85.2
Social services	36	8.8
Essential services	20	4.9
**Online class activities**		
Yes	358	87.1
No	17	4.1
No response	36	8.8
**Attempts to take licit/illicit drugs?**		
Never	372	90.5
Not often	21	5.1
**Policy violation**		
No	384	93.4
Yes	27	6.6

A Mann–Whitney U-test was performed to analyse students’ psychological concerns during lockdown. An independent hypothesis on equal probabilities of all items in the PSS and other parameters were excluded by the Chi–Square test, the results of which were highly significant. A null hypothesis was accepted with no difference regarding fear of COVID-19, and a non-significant difference (p > 0.001) was found for gender. Of the sample, females experienced more stress (2.36 ± 1.31) than males (2.32 ± 2.01). In addition, female students are more worried (mean = 3.03; p < 0.001) about their studies than male students (mean = 2.61; p < 0.001).

### PSS influencing factors and their demographic comparison

**Figure 1:**
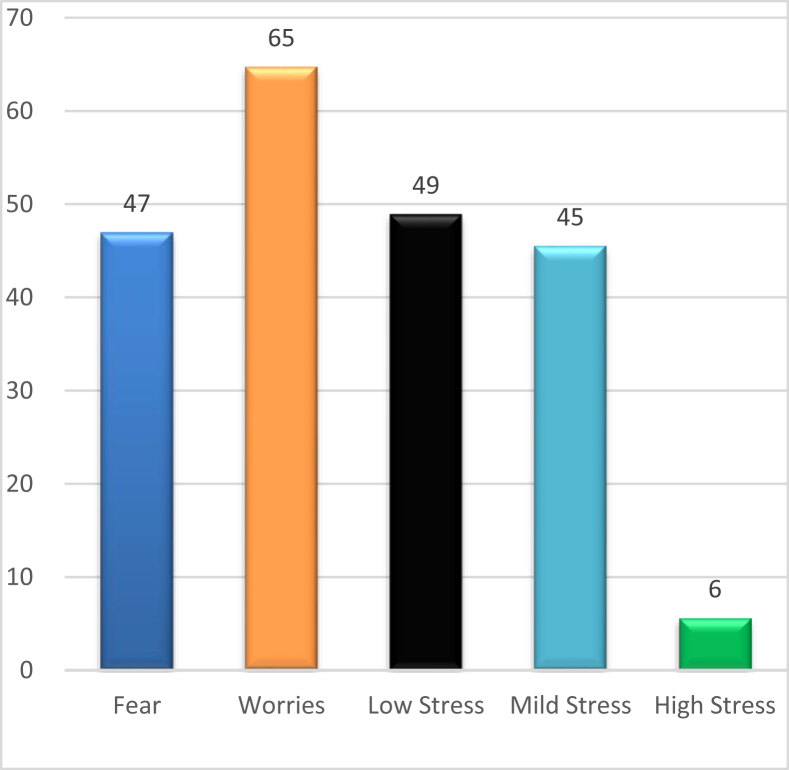
Distribution of students' mental state.

Based on the cumulative PSS score, a significant percentage of the sample have high and mild stress, although many have a low level of stress as well. The responses to the self-reported questionnaire were analysed to identify the level of fear and concerns due to COVID-19 and online classes, as shown in Figure 1. The most important factor increasing students’ level of stress was identified using RII. With [P1: Online class] as the major hurdle (RII = 0.67) and [P3: Self-management (RII = 0.65)] followed by other essentials like food and medicine, and the similar approach was applied to calculate the understanding of WHY. The most often [WHY2: Greater vulnerability to stressful life-event elicited depressive symptoms] and [WHY4: Failure to accept E-learning] were ranked highest, while other factors were not found to be significant. Considering these factors, students with mild to high stress can be classified in the 18–25 year age group (Mean ± SD = 2.0141 ± 0.610; CI = 1.95–2.07).

PSS is a multi-item questionnaire that includes positive items like ‘how often have you felt confident about your ability to handle your problems’ and negative items like ‘how often have you found that you could not cope with all the things that you had to do?’ Therefore, to identify the sum of the impact of the variables, a factor analysis of the 10-item PSS questions was performed using a Principal Component Analysis (PCA) and Varimax with the Kaiser normalisation rotation method (KMO). KMO and Bartlett's Test (0.81) were conducted before the factor analysis. The analysis categorised the 10 points into 2 major components: perceived positive impact (PSS4, PSS5, PSS7, PSS8) with factor (−0.297, −0.065, −0.336, −0.231) and perceived negative impact of the factor ranking. The final positive and negative PSS items are correlated (0.967, −0.256) between the two categories.

To understand the association of attitude variables with the PSS, a correlation analysis was performed. A strong positive relationship with the problems of P1: Online classes (r = 0.192, p < .001) and P3: Self-management (r = 0.237, p < 0.001) was observed, as shown in Table 3. The table also highlights the relationship between the PSS values and reasons behind perceiving WHY these stress levels are perceived.

**Table 3:** Correlation between PSS and attitude variables.

		P1	P3	WHY2	WHY4
P1	Correlation Coefficient	0.112∗			
	Sig. (2-tailed)	0.012			
P3	Correlation Coefficient	0.121∗	1.000		
	Sig. (2-tailed)	0.014			
WHY2	Correlation Coefficient	0.064	0.161∗∗	1.000	
	Sig. (2-tailed)	0.193	0.001		
WHY4	Correlation Coefficient	0.418∗∗	0.237∗∗	0.285∗∗	1.000
	Sig. (2-tailed)	0.000	0.000	0.000	
PSS scale of Mild to High Stress	Correlation Coefficient	0.192∗∗	0.237∗∗	0.329∗∗	0.325∗∗

An asterisk indicates significance at p < 0.05.

A one-way ANOVA with significant model fit (p < 0.05) and goodness of fit (Pearson Significance: p > 0.05) was obtained through the comparison of PSS by age group and major hurdles in terms of P1 and P3 along with factors WHY2 & WHY4. Table 4 shows the significant F values and mean square values.

**Table 4:** One-way ANOVA for factors influencing the PSS scale.

Predictors	Items	F	Mean Square	df	p
**P1:** Hurdle of online classes	Never	14.829	491.717	2	<0.001
	To some extent				
**P3:** Self-management	Too much	26.877	844.692	2	<0.001
**WHY 2:** Greater vulnerability to a stressful life	Never	22.432	656.677	4	<0.001
	Almost never				
**WHY 4:** Failure to accept E-learning	Sometimes	16.077	496.064	4	<.0001
	Fairly often				
	Very often				
**Age Group**	<18				
	18–25	1.038	0.367	2	>0.05
	>26				

A multiple ordinal logistic regression analysis was performed with stress level as the dependent variable, and age and relative hurdles (P1, P3) as covariate factors. A higher level of stress was associated with students in the age group 18–25 years with the goodness of fit to observed data (χ2 = 138.378, p < 0.001). The tests on parallel lines were insignificant, and pseudo R square values indicated a 35% variability. Table 5 provides the results of the regression analysis. The logistic regression indicates that people with more anxiety towards online classes (P1) experience mild to high stress on the PSS (OR = 2.168, 95% CI = 0.332–6.691). The instability due to a problem in managing the situation (P3) by students (OR = 23.473, 95% CI = 1.828–301.490) is also a factor that increases the level of stress. In addition, the factors identified by the PCA and ANOVA are failure to accept E-learning (OR = 13.256, 95% CI = 2.578–68.165) and greater vulnerability to stressful life events (OR = 0.752, 95% CI = 2.425–310.642), which are also relative risk factors.

**Table 5:** Logistic regression to estimate the prevalence of stress among students.

PSS_Grp^a^		SE	Sig.	Exp(B)	95% CI
LOW	Age_grp	1.043	0.206	0.267	[0.035–2.067]
	[P1–P3]	0.945	0.203	3.329	[0.522–21.232
		0.716	**0.000**	3.826	[0.941–15.564
		0.786	0.091	3.775	[0.809–17.625
		0.649	0.145	2.573	[0.721–9.174
	[WHY2 & WHY4]	1.231	**0.000**	101.689	[9.109–1135.179
		1.542	0.000	365.327	[17.797–7499.227
		1.293	**0.001**	75.197	[5.962–948.369
		1.304	0.105	8.296	[0.644–106.837
		1.011	0.128	4.652	[0.641–33.743]
		1.032	0.108	5.257	[0.696–39.707]
		1.303	**0.000**	23.473	[1.828–301.490]
		0.976	0.672	0.662	[0.098–4.484]
Mild	Age_grp	1.039	0.191	0.257	[0.033–1.968]
	[P1–P3]	0.932	0.408	2.162	[0.348–13.436]
		0.691	0.243	2.239	[0.578–8.675]
		0.766	0.603	1.490	[0.332–6.691]
		0.621	0.494	1.529	[0.453–5.167]
	[WHY2 & WHY4]	0.758	**0.000**	6.792	[1.538–29.987]
		1.192	**0.000**	24.101	[2.330–249.291]
		0.835	**0.000**	13.256	[2.578–68.165]
		0.837	0.833	1.193	[0.231–6.147]
		0.952	0.393	2.255	[0.349–14.571]
		0.959	0.214	3.292	[0.503–21.560]
		1.238	**0.000**	27.446	[2.425–310.642]
		0.835	0.887	1.126	[0.219–5.790]

Bold value presents parameters with no significance.

a The reference category is high; Nagelkerke pseudo R^2^: 0.34.

## Discussion

The study was conducted on Indian students during the COVID-19 outbreak to assess the factors associated with psychological disorders during a pandemic situation, stress in particular. The results show that fear of vulnerability, self-management, and failure to accept virtual learning impact the PSS score. Common individual behavioural effects like anxiety, stress, depression, anger, and post-traumatic stress are socially available disorders that affect students.^16^ The information and perseverance needed to manage these are influenced by additional attitude factors including fear and worries along with violation of guidelines, high pressure, and technical glitches during academic activities. All these factors affect students’ psychological behaviour and may lead to an uncontrollable situation.

Some studies highlighted that any pandemic has its course of completion, but leaves survivors with distress and associated factors like poverty, anxiety, and fear.^17^ Therefore, the authors assessed the level of stress among the student fraternity its influencing factors. Stress may even lead to some losing their lives. Moreover, a student who at a young age is more vulnerable to such a traumatic event may ultimately do exceptionable things. The survey indicated that about 25% of students are negatively affected by the outbreak and have experienced an above average level of stress. Of these students, 6% have experienced severe stress, and around 45% mild stress.

The items in the PSS were found to be significantly related to the factors that led to stress among the students in this study. The results of the correlation analysis showed that factors such as being unable to cope with the new paradigm of teaching-learning and ability to withstand vulnerability to situations like the recent pandemic are positively related. This suggests a change in the existing curriculum to ensure an appropriate fit with the online mode of teaching as well as the provision of enhanced counselling and guidance regarding the current situation. However, consuming alcohol and violation of policies are negatively correlated. With limited resources available concerning the impact on students’ life because of COVID-19, the present study also found the prevalence of stress due to the pandemic. The one-way ANOVA and multiple logistic regression confirmed the association of the PSS with routine academic studies and confinement due to COVID-19. Previous studies reported that mental and psychological episodes such as stress and fear affect the efficacy of life.^12^^,^^18^

Aligned with the hypothesis of this study, selfmanagement problems, ongoing academic activities, and the non-opening of educational institutions were all related to stress. Similar studies by^7^^,^^19^ on Chinese students also reported a psychological imbalance among university students due to the COVID-19 outbreak. An Italian study^20^ sought to understand how students are coping with the situation. In some situations, parents are also stressed by the payment of tuition fees after losing their jobs, which exacerbates students’ stress.^12^ Social support during this health emergency is thus crucial.^21^^,^^22^ In this study, students reported their interest in engaging with social activities that would help them overcome their fear and anxiety.

College students’ stress regarding COVID-19 may be related to the effect of the virus on their studies and not being able to handle the consequences of infection.^23^ On the other hand, their stress may have been caused by gradually losing attention in their online classes during the confinement period. It is known that stress and anger may lead to other negative psychological behaviour and mental illness.^24^ This indicates that the increasing number of days in lockdown and other government policies could cause students to worry about their education, growth, and careers, which further increases their anxiety and fear.^25^^,^^26^ Furthermore, it has been seen that in addition to a fear of COVID-19, students are also worried about their studies, especially female students.^27^^,^^28^

## Conclusion

The study focused on the prevalence of stress among students due to the closure of educational institutions and prolonged online teaching and learning. Students are worried about their studies and the difficulties they experience in managing themselves during the pandemic. The study found that female students are more concerned about their academic activities, and that students aged 18–25 years are more vulnerable to the impact of lockdown. They are stressed because of the inability to accept the paradigm shift in academic activities and prolonged period of COVID-19 restrictions. The intention behind the present work was to provide early evidence of disruptive episodes in terms of stress due to the closure of universities or colleges that impacts the general health of the people living in such confinement situations.

## Recommendations

During this unwanted pandemic situation, people are suffering from mental stress and students are among the worst-hit groups.^29^ Since the outbreak, their studies have been hampered because all educational institutions were closed and limited to virtual classes. Specifically, it is recommended that for engineering or medical students, both oral sessions and practical knowledge are important. However, confinement at home has meant that practical sessions are not taking place, leading to a lack of motivation regarding academic studies. These factors result in psychological pressure including depression, stress, phobia, fear, social disconnection, and so on.

## Source of funding

This research did not receive any specific grant from funding agencies in the public, commercial, or not-for-profit sectors.

## Conflicts of interest

The authors have no conflicts of interest to declare.

## Ethical approval

This article does not contain any experimental studies with human participants or animals performed by any of the authors. Ethical approval was exempted.

## Authors' contributions

BC, LMG, and MM: Conceived and designed the study, conducted research, provided research materials, and collected and organised the data. BC, LMG: Analysed and interpreted the data. BC, GB: Wrote the initial and final draft of the article and provided logistic support. All authors have critically reviewed and approved the final draft and are responsible for the content and similarity index of the manuscript.
